# Mercury(II) halide complex of *cis*-[(^*t*^BuNH)(Se)P(μ-N^*t*^Bu)_2_P(Se)(NH^*t*^Bu)]

**DOI:** 10.1107/S205698902400937X

**Published:** 2024-10-08

**Authors:** Troy Selby-Karney, Kalpana Sampath, Kuppuswamy Arumugam, Chandru P. Chandrasekaran

**Affiliations:** a Department of Chemistry and Biochemistry Lamar University, 4400 MLK Blvd Beaumont Texas 77710 USA; b Department of Chemistry, Wright State University, 3640 Colonel Glenn Hwy., Dayton, OH 45435, USA; University of Missouri-Columbia, USA

**Keywords:** crystal structure, cyclo­diphosphaza­nes, mercury(II) halide, *tert-*butyl­amido

## Abstract

The mercury(II) halide complex [1,3-di-*tert*-butyl-2,4-bis­(*tert*-butyl­amino)-1,3,2λ^5^,4λ^5^-di­aza­diphosphetidine-2,4-diselone-κ^2^*Se*,*Se*′]di­iodido­mercury(II)*N*,*N*-di­methyl­formamide monosolvate, [HgI_2_(C_16_H_38_N_4_P_2_Se_2_)]·C_3_H_7_NO or (**1**)HgI_2_, **2**, containing *cis*-[(^*t*^BuNH)(Se)P(μ-N^*t*^Bu)_2_P(Se)(NH^*t*^Bu)] (**1**) was synthesized and structurally characterized. The crystal structure of **2** confirms the chelation of chalcogen donors to HgI_2_ with a natural bite angle of 112.95 (2)°. The coordination geometry around mercury is distorted tetra­hedral as indicated by the τ_4_ geometry index parameter (τ_4_ = 0.90). In the mercury complex, the exocyclic *tert*-butyl­amido substituents are arranged in an (*endo, endo*) fashion, whereas in the free ligand (**1**), the exocyclic substituents are arranged in an (*exo, endo*) pattern. Compound **2** displays non-classical N—H⋯O hydrogen-bonding inter­actions with the solvent *N*,*N*-di­methyl­formamide. These inter­actions may introduce geometrical distortion and deviation from an ideal geometry. An isostructural HgBr_2_ analogue containing *cis*-[(^*t*^BuNH)(S)P(μ-N^*t*^Bu)_2_P(S)(NH^*t*^Bu)] was also synthesized and structurally characterized, CIF data for the compound being presented as supporting information.

## Abstract 2

The crystal structure of a mercury(II) halide complex containing bis­(*tert-*butyl­amido)­cyclo­diphosphazane ligand with an unusual chelation mode is reported. The mol­ecular structure features weak N—H⋯O inter­actions that propagate and link the mol­ecules into a three-dimensional structure.

## 1. Chemical context

**Figure 1:**
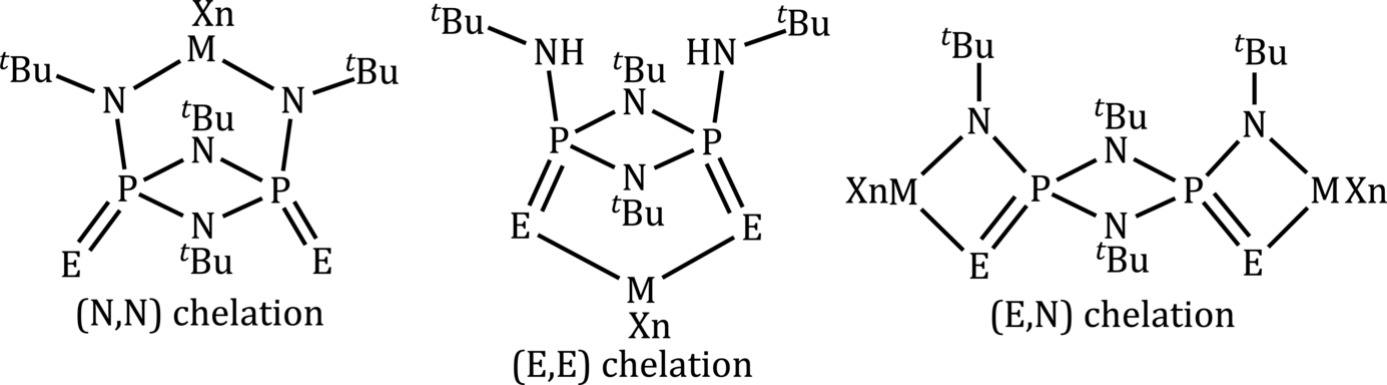
Possible coordination modes of bis­(amido)­cyclo­diphosph(V)aza­nes.

Stable four-membered rings containing phospho­rus and nitro­gen with the general formula, [(*R*)P(μ-N^*t*^Bu)_2_P(*R*)] (*R* = alkyl or ar­yl), are commonly referred to as cyclo­diphosphaza­nes. They have been used as building blocks to construct inter­esting macrocycles and polymers (Balakrishna, 2016 ▸). These macrocycles are formed by taking advantage of the *cis* orientation of the substituents and the lone pair available on the phospho­rus atom (Balakrishna, 2016 ▸; Bashall *et al.*, 2002 ▸). The bis­(amido)­cyclo­diphosphazane and its P^V^ analogue have been used as a versatile framework to stabilize main-group elements and transition metals (Stahl, 2000 ▸; Briand *et al.*, 2002 ▸). The bis­(amido)­cyclo­diphosph(V)azane, *cis*-{[(*R*)NH](*E*)P(μ-N^*t*^Bu)_2_P(*E*)[NH(*R*)]} [*E* = O, S, Se, N(*R*); *R* = alkyl or ar­yl] and its di-anionic derivatives exhibit three unique coordination modes as shown in Fig. 1 ▸. These ligands are capable of bonding to metals and non-metals *via* (*N*,*N*), (*E*,*E*) or (*N*,*E*) chelation modes. The (*N*,*E*) chelation mode is the most frequently observed because of the rigidity and planarity of the four-membered P_2_N_2_ ring. More importantly, the (*N*,*N*) and (*E*,*E*) chelation modes demand large bite angles, and large-size metal ions are well suited for these coordination modes. In 2001, Chivers *et al.* (2001 ▸) reported (*S*,*S*) chelation of *cis*-[(^*t*^BuN)(S)P(μ-N^*t*^Bu)_2_P(S)(N^*t*^Bu)] to the Pt^II^ center with a bite angle of 99.57 (13)°. Recently, we have reported (*Se*,*Se*) chelation of *cis*-[(^*t*^BuNH)(Se)P(μ-N^*t*^Bu)_2_P(Se)(NH^*t*^Bu)] by a Pd^II^ complex with a bite angle of 110.54 (1)° (Bonnette *et al.*, 2018 ▸). It is evident from these examples that the (*E*,*E*) coordination mode prefers large metal cations. Mercury ions have been well documented to have an affinity towards sulfur and selenium atoms, and accounting for its larger size, we set out to explore the coord­ination chemistry of bis­(amido)­cyclo­diphosph(V)azane ligands with mercury(II) halide. Herein, we report the synthesis and solid-state structure of an HgI_2_ coordination complex with *cis*-[(^*t*^BuNH)(Se)P(μ-N^*t*^Bu)_2_P(Se)(NH^*t*^Bu)] (**1**), and the results are presented below. An isostructural HgBr_2_ analogue was also synthesized and structurally characterized. The CIF data for the compound are presented as supporting information.
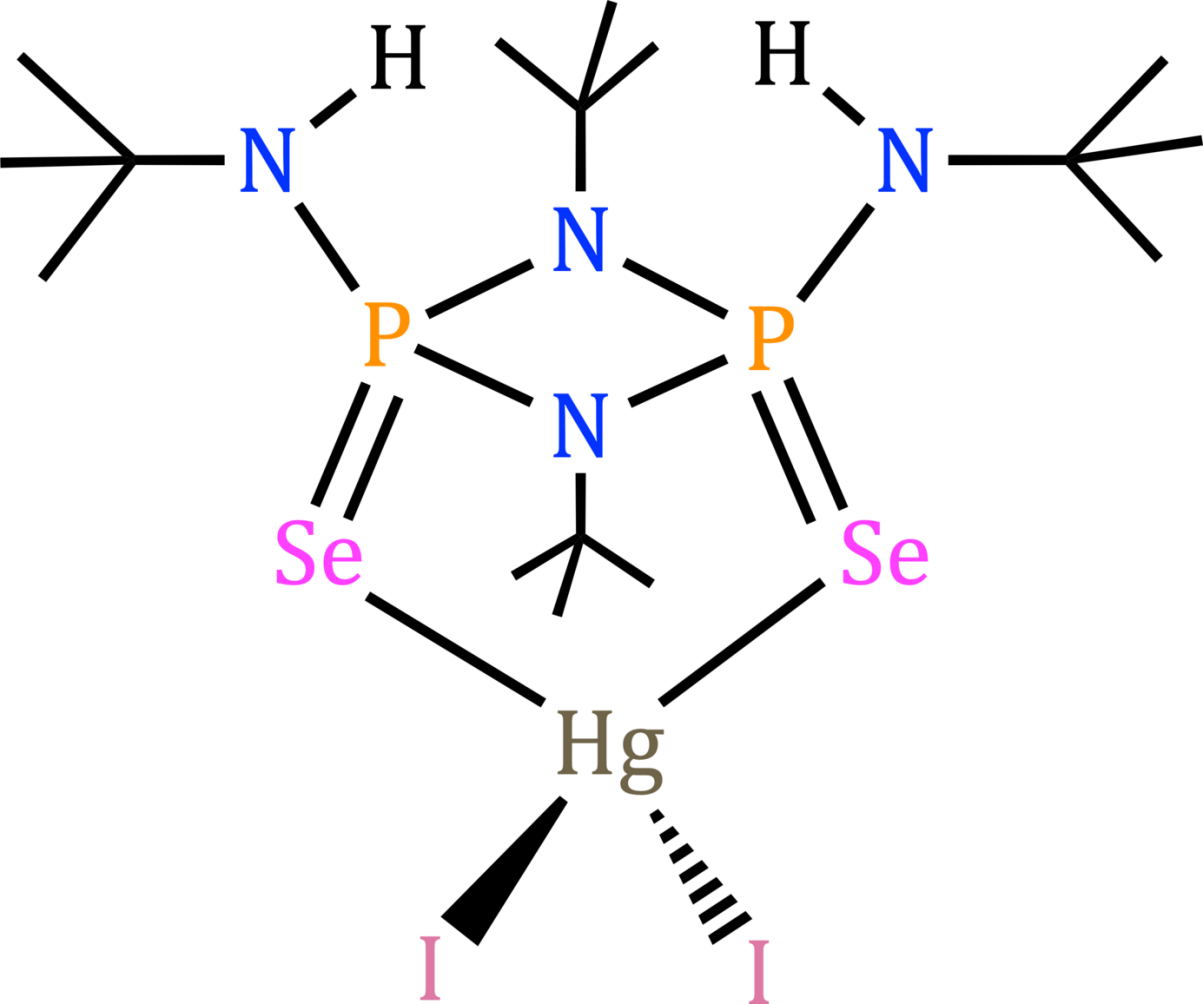


## 2. Structural commentary

**Figure 2:**
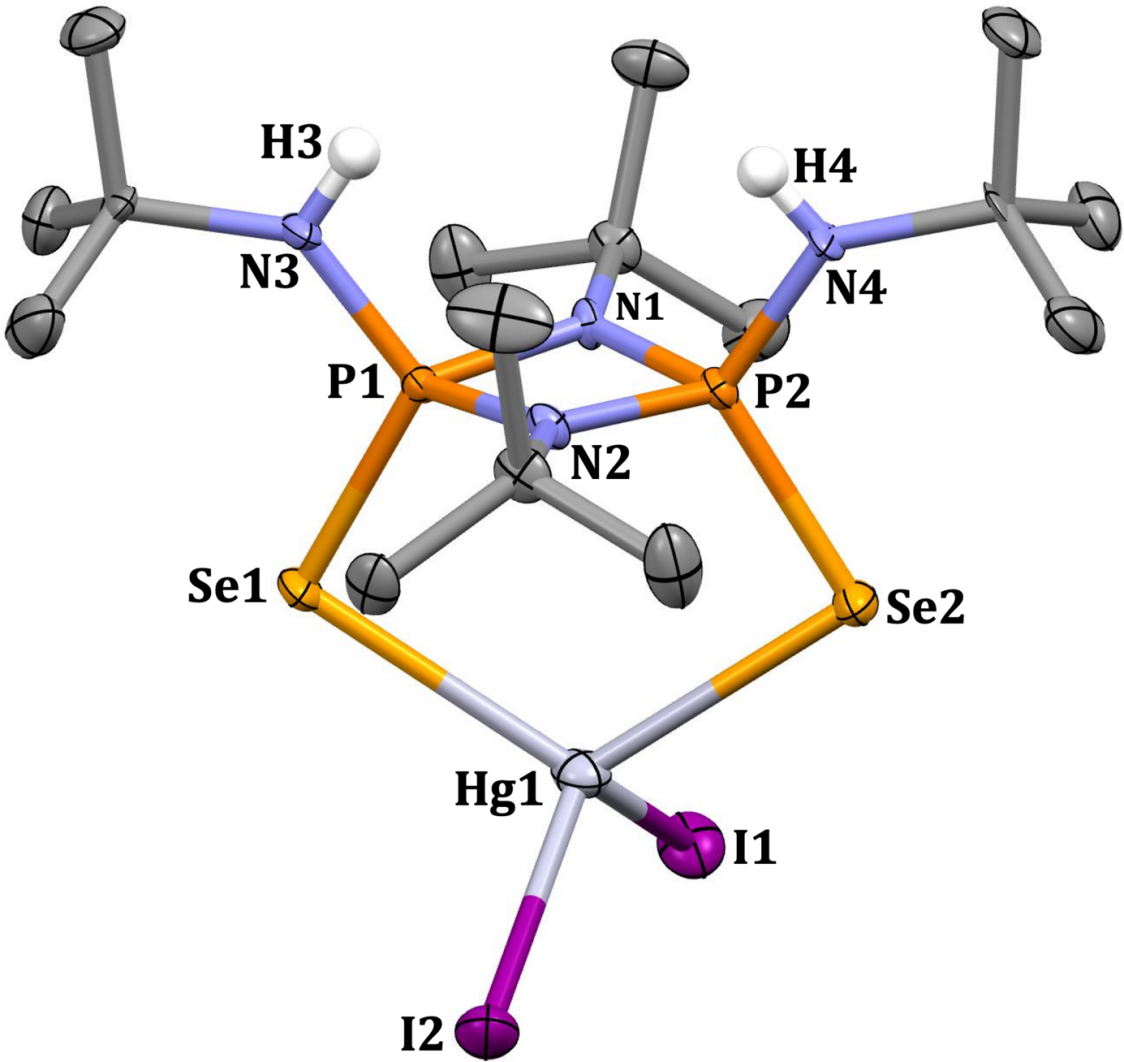
Displacement ellipsoid plot for **2** (50% probability level). The DMF solvent mol­ecule and all the hydrogen atoms are omitted for clarity, except for those at N3 and N4.

Compound **2** crystallizes in the monoclinic crystal system in space group *P*2_1_/*n*. The mol­ecular structure of **2** is illustrated in Fig. 2 ▸. The crystal structure confirms the chelation of **1** through selenium donors to stabilize the HgI_2_ moiety, with an Se1—Hg1—Se2 natural bite angle of 112.95 (2)°. The coordination geometry around the mercury atom is distorted tetra­hedral, as indicated by the parameter τ_4_ = 0.90. The geometry index τ_4_ was developed by Okuniewski and co-workers to distinguish various four-coordinate geometries (Okuniewski *et al.*, 2015 ▸; Yang *et al.*, 2007 ▸) with τ_4_ = 0 for a square-planar geometry, 0.24 for seesaw, and 1 for a tetra­hedral geometry. The Hg—Se [Hg1—Se1 = 2.7508 (5) Å; Hg1–Se2 = 2.7835 (6) Å], and Hg—I [Hg1—I1 = 2.7290 (4) Å; Hg1–I2 = 2.7409 (4) Å] bond distances are within the typical ranges reported for the HgI_2_ complexes with selenium ligands (Palmer & Parkin, 2015 ▸). In complex **2**, the P1—Se1 and P2—Se2 bonds [2.1260 (13) and 2.1302 (12) Å, respectively] are slightly elongated compared to the P—Se bond [2.078 (1) Å] in the uncoordinated ligand **1**. The four-membered P_2_N_2_ ring in complex **2** is slightly puckered, as indicated by the angle subtended by the planes N1/P1/N2 and N1/P2/N2 [8.7 (3)°]. The corresponding dihedral angle for the uncoordinated ligand is 3.73 (2)° (Hill *et al.*, 1994 ▸).

## 3. Supra­molecular features

**Figure 3:**
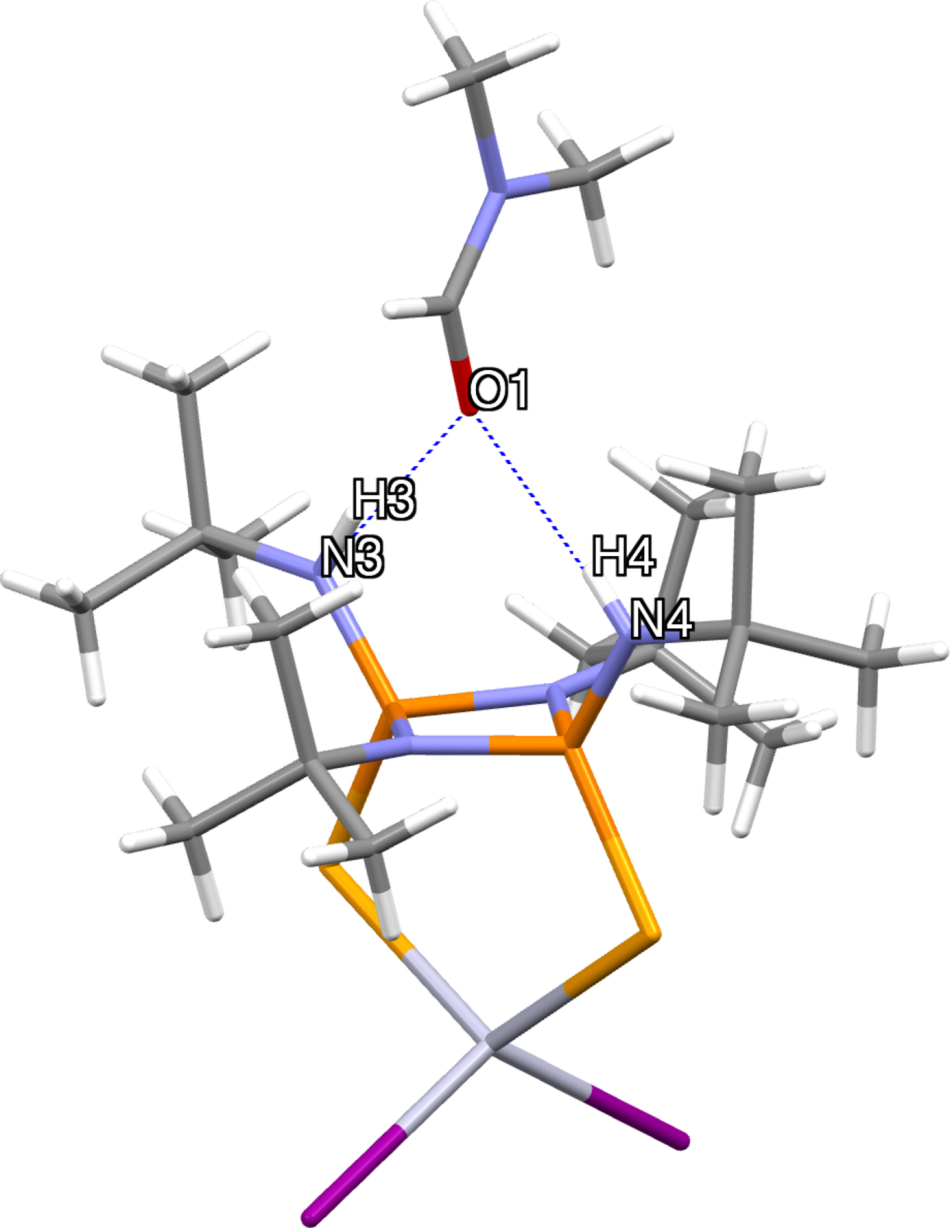
Hydrogen-bonding inter­actions (Table 1 ▸) between the complex and the DMF solvent mol­ecule in the crystal.

**Figure 4:**
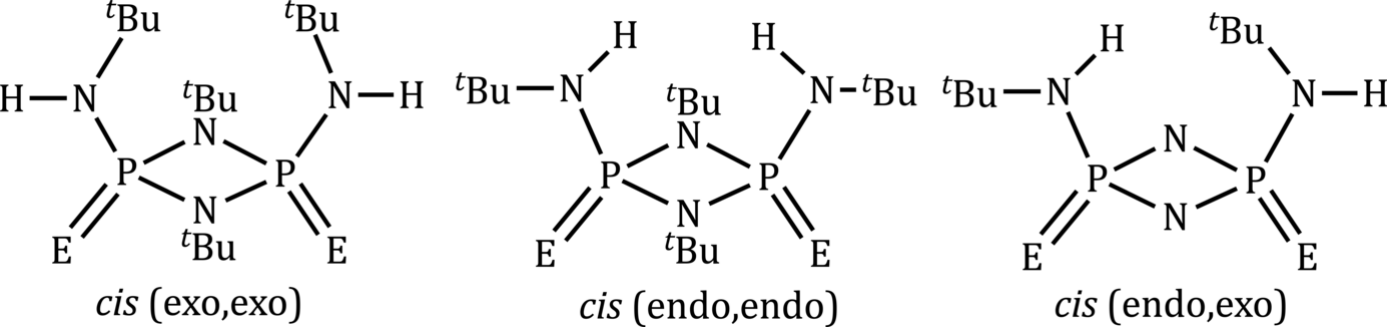
Possible conformational isomers for *cis*-bis­(amido)­cyclo­diphosph(V)aza­nes.

In the crystal of **2**, the N—H functional groups present in bis­(*tert*-butyl­amido)­cyclo­diphosph(V)azane and oxygen from the DMF solvent mol­ecule are involved in N—H⋯O hydrogen-bonding inter­actions (Fig. 3 ▸, Table 1 ▸). Three different conformational isomers are feasible for the *cis-*bis­(amido)­cyclo­diphosph(V)azane with respect to the relative orientations of the exocyclic nitro­gen substituents (Fig. 4 ▸). In **2**, the exocyclic substituents are arranged in a (*endo, endo*) fashion, whereas in ligand **1** they are arranged in an (*exo*, *endo*) orientation (Hill *et al.*, 1994 ▸; Chivers *et al.*, 2002 ▸). The conformational change in the coordination sphere of **2** may result from the formation of inter­molecular inter­actions. A similar conformational change influenced by hydrogen-bonding inter­actions has been previously reported (Chandrasekaran *et al.*, 2011 ▸).

## 4. Database survey

A search of the Cambridge Structural Database (CSD Data, March 2024; Groom *et al.*, 2016 ▸) gave the following hits for *cis*-{[(*R*)_*n*_CN](Se)P[(*R*)_*n*_N]_2_P(Se)[NC(*R*)_*n*_]}: di­chlorido­[1,3-di-*tert*-butyl-2,4-bis­(*tert*-butyl­amino)-1,3,2,4-di­aza­diphosphet­id­ine-2,4-diselone-Se,Se’]palladium(II)} (Bonnette *et al.*, 2018 ▸; CCDC No. 1549758), bis­[μ-(2,4-bis­(*tert*-butyl­amido)-1,3-bis­(*tert*-but­yl)-2,4-di­seleno-2,4-diphosphetidine)]hexa­kis­(tetra­hydro­furan)­tetra­potassium, (Chivers *et al.*, 2001 ▸; CCDC No. 142628), bis-*N*,*N*′,1,3-tetra-*tert*-butyl-1,3,2,4-di­aza­diphosphet­id­ine-2,4-di­amine 2,4-bis­(selenide)]silver(I) tri­fluoro­meth­ane­sulfonate (Knight & Woollins, 2016 ▸; CCDC No. 1042705) and [1,3-di-*tert*-butyl-2,4-bis­(*tert*-butyl­amino)-1,3,2,4-di­aza­diphosphetidine-2,4-diselone]bis­(tri­phenyl­phosphine)palladium bis­(tetra­fluoro­borate) di­chloro­methane solvate (Plajer *et al.*, 2020 ▸; CCDC No. 1890520). The P=Se and P—N bond distances for **2** are in agreement with those in the above compounds.

## 5. Synthesis and crystallization

Synthesis of *cis*-[HgI_2_(**1**)] (**2**):

A di­chloro­methane (10 mL) solution of *cis*-[(^*t*^BuHN)(Se)P(μ-^*t*^BuN)_2_P(Se)(NH^*t*^Bu)] (**1**) (100 mg, 0.197 mmol) was added dropwise over an aceto­nitrile (5 mL) solution of HgI_2_ (88 mg; 0.197 mmol) under an N_2_ atmosphere at ambient temperature. The resulting reaction mixture was stirred for 4 h at 295 K. The solution was then concentrated to nearly 5 mL and stored at 248 K for a day to afford an analytically pure white microcrystalline product. Yield: 91% (172 mg). X-ray quality crystals are obtained by slow evaporation from a DMF solution at room temperature, m.p. 465–467 K. ^1^H NMR (400 MHz, DMSO-*d*_6_): 1.44 (*s*, 18H, ^*t*^Bu), 1.58 (*s*, 18H, ^*t*^Bu), 2.58 (*br s*, 2H, NH). IR (cm^−1^): 3200 (*br w*), 2975 (*w*), 1462 (*w*), 1388 (*w*), 1366 (*m*), 1222 (*w*), 1183 (*m*), 1030 (*vs*), 899 (*s*), 851 (*w*), 731 (*m*), 678 (*m*). Absorption spectrum [DMSO; λ_max_, nm (ɛ_M_, *M*^−1^ cm^−1^)]: 270 (13947). Analysis calculated for C_16_H_38_N_4_P_2_Se_2_HgI_2_: C, 20.00; H, 3.99; N, 5.83. Found: C, 20.26; H, 4.47; N, 5.98.

## 6. Refinement

Crystal data, data collection and structure refinement details are summarized in Table 2 ▸. Methyl (CH_3_) hydrogen atoms were treated as a rotating group and added using the riding-model approximation to the carbon atom to which they are attached [C—H = 0.98 Å with *U*_iso_(H) = 1.5*U*_eq_(CH_3_).

## 7. Data for isostructural HgBr_2_ complex

Synthesis and spectroscopic data for an isostructural HgBr_2_ with *cis*-[(^*t*^BuHN)(S)P(μ-^*t*^BuN)_2_P(S)(NH^*t*^Bu)] are presented below. Spectroscopic analysis and single-crystal structure determination strongly support these are isostructural complexes. For more information regarding solid-state structure determination, please refer to CCDC: 2380829. The CIF data for this compound is available in the supporting information

A di­chloro­methane (10 mL) solution of *cis*-[(^*t*^BuHN)(S)P(μ-^*t*^BuN)_2_P(S)(NH^*t*^Bu)] (**1**) (100 mg, 0.24 mmol) was added dropwise over an aceto­nitrile (5 mL) solution of HgBr_2_ (87.4 mg; 0.24 mmol) under an N_2_ atmosphere at ambient temperature. The resulting reaction mixture was stirred for 4 h at 295 K. The solution was then concentrated to nearly 5 mL and stored at 248 K for a day to afford an analytically pure white microcrystalline product. Yield: 83% (156 mg). X-ray quality crystals were obtained by slow evaporation from DMF solution at room temperature, m.p. 483–485 K. ^1^H NMR (400 MHz, DMSO-*d*_6_): 1.45 (*s*, 18H, ^*t*^Bu), 1.59 (*s*, 18H, ^*t*^Bu), 2.54 (*br s*, 2H, NH). IR (cm^−1^): 3194 (*br m*; N-H), 2977 (*w*), 1471 (*w*), 1391 (*w*), 1369 (*m*), 1225 (*w*), 1185 (*s*), 1046 (*vs*), 907 (*m*), 852 (*m*), 745 (*s*), 705 (*m*). Absorption spectrum [DMSO; λ_max_, nm (ɛ_M_, *M*^−1^ cm^−1^)]: 282 (17054). Analysis calculated for C_16_H_38_N_4_P_2_S_2_HgBr_2_: C, 24.86; H, 4.95; N, 7.25; S, 8.30. Found: C, 25.03; H, 5.08; N, 7.75; S, 8.22.

## Supplementary Material

Crystal structure: contains datablock(s) I. DOI: 10.1107/S205698902400937X/ev2009sup1.cif

Structure factors: contains datablock(s) I. DOI: 10.1107/S205698902400937X/ev2009Isup2.hkl

CIF Data for isomorphous HgBr2 complex. DOI: 10.1107/S205698902400937X/ev2009sup3.txt

CCDC reference: 2386028

Additional supporting information:  crystallographic information; 3D view; checkCIF report

## Figures and Tables

**Table 1:** Hydrogen-bond geometry (Å, °)

*D*—H⋯*A*	*D*—H	H⋯*A*	*D*⋯*A*	*D*—H⋯*A*
N3—H3⋯O1	0.82 (6)	2.18 (6)	2.986 (6)	165 (4)
N4—H4⋯O1	0.79 (5)	2.27 (5)	3.051 (5)	169 (6)

**Table 2:** Experimental details

Crystal data	
Chemical formula	[HgI_2_(C_16_H_38_N_4_P_2_Se_2_)]·C_3_H_7_NO
*M*_r_	1033.85
Crystal system, space group	Monoclinic, *P*2_1_/*n*
Temperature (K)	300
*a*, *b*, *c* (Å)	9.1747 (8), 17.3698 (13), 20.841 (2)
β (°)	101.049 (3)
*V* (Å^3^)	3259.7 (5)
*Z*	4
Radiation type	Mo *K*α
μ (mm^−1^)	8.97
Crystal size (mm)	0.53 × 0.28 × 0.26

Data collection	
Diffractometer	Bruker *SMART* X2S
Absorption correction	Multi-scan (*SADABS*; Krause *et al.*, 2015 ▸
*T*_min_, *T*_max_	0.309, 0.746
No. of measured, independent and observed [*I* > 2σ(*I*)] reflections	45600, 9576, 6956
*R*_int_	0.077
(sin θ/λ)_max_ (Å^−1^)	0.725

Refinement	
*R*[*F*^2^ > 2σ(*F*^2^)], *wR*(*F*^2^), *S*	0.040, 0.089, 1.00
No. of reflections	9576
No. of parameters	310
H-atom treatment	H atoms treated by a mixture of independent and constrained refinement
Δρ_max_, Δρ_min_ (e Å^−3^)	1.91, −1.92

Computer programs: *APEX2* and *SAINT* (Bruker, 2016 ▸), *SHELXT2018/2* (Sheldrick, 2015*a* ▸), *SHELXL2019/1* (Sheldrick, 2015*b* ▸), *Mercury* (Macrae *et al.*, 2020 ▸) and *OLEX2* (Dolomanov *et al.*, 2009 ▸).

## supplementary crystallographic information

### [1,3-Di-tert-butyl-2,4-bis(tert-butylamino)-1,3,2λ5,4λ5-diazadiphosphetidine-2,4-diselone-κ2Se,Se']diiodidomercury(II) N,N-dimethylformamide monosolvate Crystal data

**Table:**

[HgI_2_(C_16_H_38_N_4_P_2_Se_2_)]·C_3_H_7_NO	*F*(000) = 1944
*M**_r_* = 1033.85	*D*_x_ = 2.107 Mg m^−^^3^
Monoclinic, *P*2_1_/*n*	Mo *K*α radiation, λ = 0.71073 Å
*a* = 9.1747 (8) Å	Cell parameters from 8829 reflections
*b* = 17.3698 (13) Å	θ = 2.3–26.8°
*c* = 20.841 (2) Å	µ = 8.97 mm^−^^1^
β = 101.049 (3)°	*T* = 300 K
*V* = 3259.7 (5) Å^3^	Prism, colorless
*Z* = 4	0.53 × 0.28 × 0.26 mm

### [1,3-Di-tert-butyl-2,4-bis(tert-butylamino)-1,3,2λ5,4λ5-diazadiphosphetidine-2,4-diselone-κ2Se,Se']diiodidomercury(II) N,N-dimethylformamide monosolvate Data collection

**Table:**

Bruker SMART X2S diffractometer	6956 reflections with *I* > 2σ(*I*)
ω scans	*R*_int_ = 0.077
Absorption correction: multi-scan (SADABS; Krause *et al.*, 2015)	θ_max_ = 31.0°, θ_min_ = 2.6°
*T*_min_ = 0.309, *T*_max_ = 0.746	*h* = −12→13
45600 measured reflections	*k* = −24→19
9576 independent reflections	*l* = −28→29

### [1,3-Di-tert-butyl-2,4-bis(tert-butylamino)-1,3,2λ5,4λ5-diazadiphosphetidine-2,4-diselone-κ2Se,Se']diiodidomercury(II) N,N-dimethylformamide monosolvate Refinement

**Table:**

Refinement on *F*^2^	Hydrogen site location: mixed
Least-squares matrix: full	H atoms treated by a mixture of independent and constrained refinement
*R*[*F*^2^ > 2σ(*F*^2^)] = 0.040	*w* = 1/[σ^2^(*F*_o_^2^) + (0.0325*P*)^2^] where *P* = (*F*_o_^2^ + 2*F*_c_^2^)/3
*wR*(*F*^2^) = 0.089	(Δ/σ)_max_ = 0.004
*S* = 1.00	Δρ_max_ = 1.91 e Å^−^^3^
9576 reflections	Δρ_min_ = −1.91 e Å^−^^3^
310 parameters	Extinction correction: *SHELXL2019/1* (Sheldrick, 2015b), Fc^*^=kFc[1+0.001xFc^2^λ^3^/sin(2θ)]^-1/4^
0 restraints	Extinction coefficient: 0.00052 (5)

### [1,3-Di-tert-butyl-2,4-bis(tert-butylamino)-1,3,2λ5,4λ5-diazadiphosphetidine-2,4-diselone-κ2Se,Se']diiodidomercury(II) N,N-dimethylformamide monosolvate Special details

**Table:**

Geometry. All esds (except the esd in the dihedral angle between two l.s. planes) are estimated using the full covariance matrix. The cell esds are taken into account individually in the estimation of esds in distances, angles and torsion angles; correlations between esds in cell parameters are only used when they are defined by crystal symmetry. An approximate (isotropic) treatment of cell esds is used for estimating esds involving l.s. planes.

### [1,3-Di-tert-butyl-2,4-bis(tert-butylamino)-1,3,2λ5,4λ5-diazadiphosphetidine-2,4-diselone-κ2Se,Se']diiodidomercury(II) N,N-dimethylformamide monosolvate Fractional atomic coordinates and isotropic or equivalent isotropic displacement parameters (Å^2^)

**Table:**

	*x*	*y*	*z*	*U*_iso_*/*U*_eq_	
Hg1	0.70533 (2)	0.77295 (2)	0.58150 (2)	0.02178 (6)	
I1	0.60217 (4)	0.91950 (2)	0.55729 (2)	0.02891 (9)	
I2	0.90720 (4)	0.72186 (2)	0.50978 (2)	0.02938 (9)	
Se1	0.49422 (5)	0.65923 (3)	0.55275 (2)	0.01821 (11)	
Se2	0.81087 (5)	0.79005 (3)	0.71512 (2)	0.01874 (11)	
P1	0.51042 (12)	0.62623 (6)	0.65210 (6)	0.0119 (2)	
P2	0.67826 (12)	0.69810 (6)	0.73987 (6)	0.0120 (2)	
N2	0.6846 (4)	0.61881 (19)	0.69491 (18)	0.0131 (8)	
N1	0.4974 (4)	0.6991 (2)	0.70322 (19)	0.0127 (7)	
O1	0.4763 (4)	0.5489 (2)	0.80940 (19)	0.0317 (9)	
N3	0.4072 (4)	0.5541 (2)	0.6635 (2)	0.0164 (8)	
H3	0.413 (5)	0.547 (3)	0.703 (3)	0.020*	
N4	0.7002 (4)	0.6801 (2)	0.81733 (19)	0.0158 (8)	
H4	0.649 (5)	0.644 (3)	0.820 (3)	0.019*	
N5	0.3773 (5)	0.4941 (2)	0.8902 (2)	0.0274 (10)	
C5	0.8046 (5)	0.5593 (3)	0.6959 (3)	0.0193 (10)	
C6	0.7986 (5)	0.5287 (3)	0.6268 (3)	0.0243 (11)	
H6A	0.704074	0.504766	0.611382	0.036*	
H6B	0.876292	0.491634	0.627173	0.036*	
H6C	0.811614	0.570583	0.598316	0.036*	
C11	0.4061 (6)	0.4524 (3)	0.5785 (3)	0.0333 (14)	
H11A	0.450455	0.488033	0.552709	0.050*	
H11B	0.343410	0.417284	0.550175	0.050*	
H11C	0.482767	0.424099	0.606659	0.050*	
C9	0.3141 (5)	0.4963 (2)	0.6196 (2)	0.0181 (10)	
C1	0.3696 (5)	0.7508 (3)	0.7113 (2)	0.0163 (9)	
C10	0.1830 (6)	0.5362 (3)	0.5759 (3)	0.0323 (13)	
H10A	0.124250	0.562406	0.602559	0.048*	
H10B	0.122930	0.498552	0.549292	0.048*	
H10C	0.219481	0.572836	0.548227	0.048*	
C13	0.7805 (5)	0.7175 (3)	0.8783 (2)	0.0187 (10)	
C4	0.4225 (5)	0.8335 (3)	0.7189 (3)	0.0261 (12)	
H4A	0.493585	0.838807	0.758917	0.039*	
H4B	0.339431	0.866754	0.719846	0.039*	
H4C	0.468055	0.847230	0.682688	0.039*	
C7	0.9542 (5)	0.5971 (3)	0.7207 (3)	0.0355 (14)	
H7A	0.969762	0.637485	0.691327	0.053*	
H7B	1.031512	0.559332	0.723061	0.053*	
H7C	0.955868	0.618207	0.763393	0.053*	
C15	0.9469 (5)	0.7146 (3)	0.8823 (3)	0.0311 (13)	
H15A	0.978069	0.662043	0.880656	0.047*	
H15B	0.995907	0.737546	0.922644	0.047*	
H15C	0.972166	0.742541	0.846240	0.047*	
C8	0.7780 (7)	0.4949 (3)	0.7420 (3)	0.0422 (16)	
H8A	0.783163	0.515386	0.785149	0.063*	
H8B	0.852543	0.455882	0.743044	0.063*	
H8C	0.681607	0.472865	0.726814	0.063*	
C3	0.2467 (5)	0.7432 (3)	0.6512 (3)	0.0314 (13)	
H3A	0.282388	0.760832	0.613343	0.047*	
H3B	0.163030	0.773861	0.656945	0.047*	
H3C	0.217140	0.690297	0.645353	0.047*	
C12	0.2550 (6)	0.4405 (3)	0.6658 (3)	0.0279 (12)	
H12A	0.336981	0.416839	0.694597	0.042*	
H12B	0.195746	0.401487	0.640602	0.042*	
H12C	0.195348	0.468331	0.691109	0.042*	
C16	0.7383 (6)	0.6705 (3)	0.9345 (2)	0.0261 (11)	
H16A	0.632584	0.671853	0.931349	0.039*	
H16B	0.785992	0.692134	0.975582	0.039*	
H16C	0.770054	0.618135	0.931728	0.039*	
C19	0.3824 (7)	0.4343 (3)	0.9396 (3)	0.0422 (16)	
H19A	0.291802	0.405187	0.930901	0.063*	
H19B	0.393780	0.457611	0.982021	0.063*	
H19C	0.464897	0.400734	0.938354	0.063*	
C14	0.7257 (6)	0.8005 (3)	0.8827 (3)	0.0302 (12)	
H14A	0.748134	0.829921	0.846829	0.045*	
H14B	0.774377	0.823051	0.923183	0.045*	
H14C	0.620304	0.800291	0.880742	0.045*	
C17	0.4744 (6)	0.4989 (3)	0.8515 (3)	0.0310 (13)	
H17	0.548158	0.461504	0.856240	0.037*	
C2	0.3118 (6)	0.7242 (3)	0.7719 (3)	0.0269 (12)	
H2A	0.281575	0.671275	0.766623	0.040*	
H2B	0.228388	0.755317	0.777098	0.040*	
H2C	0.389085	0.729280	0.809801	0.040*	
C18	0.2577 (8)	0.5492 (4)	0.8855 (4)	0.072 (3)	
H18A	0.164600	0.523685	0.870597	0.108*	
H18B	0.270631	0.588982	0.855095	0.108*	
H18C	0.258555	0.571560	0.927697	0.108*	

### [1,3-Di-tert-butyl-2,4-bis(tert-butylamino)-1,3,2λ5,4λ5-diazadiphosphetidine-2,4-diselone-κ2Se,Se']diiodidomercury(II) N,N-dimethylformamide monosolvate Atomic displacement parameters (Å^2^)

**Table:**

	*U*^11^	*U*^22^	*U*^33^	*U*^12^	*U*^13^	*U*^23^
Hg1	0.02744 (11)	0.02015 (11)	0.01887 (11)	−0.00093 (7)	0.00728 (8)	0.00364 (8)
I1	0.0387 (2)	0.02087 (18)	0.0257 (2)	0.00404 (14)	0.00268 (15)	0.00612 (14)
I2	0.02607 (17)	0.0364 (2)	0.0285 (2)	−0.00545 (14)	0.01224 (15)	−0.00543 (16)
Se1	0.0244 (2)	0.0215 (2)	0.0084 (2)	−0.00507 (19)	0.00213 (18)	0.00068 (19)
Se2	0.0234 (2)	0.0186 (2)	0.0138 (2)	−0.00661 (18)	0.00253 (19)	0.00001 (19)
P1	0.0155 (5)	0.0115 (5)	0.0088 (6)	−0.0001 (4)	0.0026 (4)	−0.0007 (4)
P2	0.0137 (5)	0.0134 (6)	0.0084 (6)	0.0007 (4)	0.0011 (4)	−0.0003 (5)
N2	0.0138 (17)	0.0145 (19)	0.0106 (19)	0.0040 (14)	0.0015 (15)	−0.0008 (15)
N1	0.0149 (17)	0.0106 (17)	0.012 (2)	0.0012 (14)	0.0005 (15)	−0.0037 (15)
O1	0.040 (2)	0.033 (2)	0.025 (2)	−0.0009 (17)	0.0146 (18)	0.0076 (17)
N3	0.025 (2)	0.016 (2)	0.009 (2)	−0.0055 (16)	0.0064 (17)	−0.0010 (16)
N4	0.023 (2)	0.019 (2)	0.0047 (19)	−0.0072 (16)	0.0006 (15)	0.0008 (16)
N5	0.039 (3)	0.026 (2)	0.018 (2)	−0.0017 (19)	0.008 (2)	0.0066 (19)
C5	0.020 (2)	0.016 (2)	0.023 (3)	0.0082 (18)	0.006 (2)	−0.001 (2)
C6	0.019 (2)	0.025 (3)	0.031 (3)	0.003 (2)	0.010 (2)	−0.013 (2)
C11	0.036 (3)	0.027 (3)	0.041 (4)	−0.010 (2)	0.018 (3)	−0.014 (3)
C9	0.021 (2)	0.014 (2)	0.021 (3)	−0.0110 (18)	0.010 (2)	−0.006 (2)
C1	0.017 (2)	0.015 (2)	0.018 (3)	0.0061 (18)	0.0039 (19)	−0.0005 (19)
C10	0.029 (3)	0.038 (3)	0.029 (3)	−0.013 (2)	0.000 (2)	−0.006 (3)
C13	0.024 (2)	0.028 (3)	0.004 (2)	−0.001 (2)	0.0003 (18)	−0.0053 (19)
C4	0.026 (3)	0.016 (2)	0.039 (4)	0.005 (2)	0.012 (2)	−0.001 (2)
C7	0.023 (3)	0.044 (3)	0.037 (4)	0.007 (2)	−0.001 (2)	−0.017 (3)
C15	0.028 (3)	0.050 (3)	0.015 (3)	−0.003 (2)	0.002 (2)	−0.001 (2)
C8	0.052 (4)	0.031 (3)	0.049 (4)	0.028 (3)	0.023 (3)	0.023 (3)
C3	0.023 (3)	0.036 (3)	0.032 (3)	0.013 (2)	−0.003 (2)	−0.010 (3)
C12	0.037 (3)	0.019 (3)	0.032 (3)	−0.008 (2)	0.017 (3)	0.002 (2)
C16	0.037 (3)	0.031 (3)	0.010 (3)	−0.001 (2)	0.005 (2)	0.003 (2)
C19	0.068 (4)	0.036 (3)	0.022 (3)	−0.008 (3)	0.007 (3)	0.012 (3)
C14	0.043 (3)	0.029 (3)	0.017 (3)	0.000 (2)	0.001 (2)	−0.004 (2)
C17	0.027 (3)	0.034 (3)	0.032 (3)	0.007 (2)	0.007 (2)	0.006 (3)
C2	0.028 (3)	0.029 (3)	0.027 (3)	0.009 (2)	0.013 (2)	0.005 (2)
C18	0.077 (5)	0.085 (6)	0.069 (6)	0.043 (4)	0.050 (5)	0.041 (5)

### [1,3-Di-tert-butyl-2,4-bis(tert-butylamino)-1,3,2λ5,4λ5-diazadiphosphetidine-2,4-diselone-κ2Se,Se']diiodidomercury(II) N,N-dimethylformamide monosolvate Geometric parameters (Å, º)

**Table:**

Hg1—I1	2.7290 (4)	C10—H10B	0.9600
Hg1—I2	2.7409 (4)	C10—H10C	0.9600
Hg1—Se1	2.7508 (5)	C13—C15	1.514 (6)
Hg1—Se2	2.7835 (6)	C13—C16	1.536 (7)
Se1—P1	2.1260 (13)	C13—C14	1.535 (7)
Se2—P2	2.1302 (12)	C4—H4A	0.9600
P1—P2	2.4893 (16)	C4—H4B	0.9600
P1—N2	1.679 (4)	C4—H4C	0.9600
P1—N1	1.674 (4)	C7—H7A	0.9600
P1—N3	1.615 (4)	C7—H7B	0.9600
P2—N2	1.673 (4)	C7—H7C	0.9600
P2—N1	1.689 (4)	C15—H15A	0.9600
P2—N4	1.619 (4)	C15—H15B	0.9600
N2—C5	1.507 (5)	C15—H15C	0.9600
N1—C1	1.511 (5)	C8—H8A	0.9600
O1—C17	1.237 (6)	C8—H8B	0.9600
N3—H3	0.83 (5)	C8—H8C	0.9600
N3—C9	1.508 (6)	C3—H3A	0.9600
N4—H4	0.79 (5)	C3—H3B	0.9600
N4—C13	1.491 (6)	C3—H3C	0.9600
N5—C19	1.457 (6)	C12—H12A	0.9600
N5—C17	1.314 (7)	C12—H12B	0.9600
N5—C18	1.444 (7)	C12—H12C	0.9600
C5—C6	1.526 (7)	C16—H16A	0.9600
C5—C7	1.520 (7)	C16—H16B	0.9600
C5—C8	1.525 (7)	C16—H16C	0.9600
C6—H6A	0.9600	C19—H19A	0.9600
C6—H6B	0.9600	C19—H19B	0.9600
C6—H6C	0.9600	C19—H19C	0.9600
C11—H11A	0.9600	C14—H14A	0.9600
C11—H11B	0.9600	C14—H14B	0.9600
C11—H11C	0.9600	C14—H14C	0.9600
C11—C9	1.519 (6)	C17—H17	0.9300
C9—C10	1.530 (7)	C2—H2A	0.9600
C9—C12	1.536 (6)	C2—H2B	0.9600
C1—C4	1.515 (6)	C2—H2C	0.9600
C1—C3	1.522 (7)	C18—H18A	0.9600
C1—C2	1.531 (7)	C18—H18B	0.9600
C10—H10A	0.9600	C18—H18C	0.9600
			
I1—Hg1—I2	116.603 (13)	H10A—C10—H10C	109.5
I1—Hg1—Se1	115.134 (15)	H10B—C10—H10C	109.5
I1—Hg1—Se2	97.310 (14)	N4—C13—C15	111.2 (4)
I2—Hg1—Se1	99.784 (14)	N4—C13—C16	105.3 (4)
I2—Hg1—Se2	115.997 (16)	N4—C13—C14	110.2 (4)
Se1—Hg1—Se2	112.950 (15)	C15—C13—C16	109.9 (4)
P1—Se1—Hg1	93.82 (3)	C15—C13—C14	111.4 (4)
P2—Se2—Hg1	93.27 (4)	C14—C13—C16	108.7 (4)
Se1—P1—P2	119.89 (6)	C1—C4—H4A	109.5
N2—P1—Se1	114.83 (14)	C1—C4—H4B	109.5
N2—P1—P2	41.95 (12)	C1—C4—H4C	109.5
N1—P1—Se1	114.57 (14)	H4A—C4—H4B	109.5
N1—P1—P2	42.48 (12)	H4A—C4—H4C	109.5
N1—P1—N2	84.07 (18)	H4B—C4—H4C	109.5
N3—P1—Se1	114.75 (16)	C5—C7—H7A	109.5
N3—P1—P2	125.36 (16)	C5—C7—H7B	109.5
N3—P1—N2	112.8 (2)	C5—C7—H7C	109.5
N3—P1—N1	112.2 (2)	H7A—C7—H7B	109.5
Se2—P2—P1	119.98 (6)	H7A—C7—H7C	109.5
N2—P2—Se2	113.51 (14)	H7B—C7—H7C	109.5
N2—P2—P1	42.15 (12)	C13—C15—H15A	109.5
N2—P2—N1	83.79 (17)	C13—C15—H15B	109.5
N1—P2—Se2	116.10 (14)	C13—C15—H15C	109.5
N1—P2—P1	42.01 (12)	H15A—C15—H15B	109.5
N4—P2—Se2	114.77 (15)	H15A—C15—H15C	109.5
N4—P2—P1	125.25 (15)	H15B—C15—H15C	109.5
N4—P2—N2	112.8 (2)	C5—C8—H8A	109.5
N4—P2—N1	112.1 (2)	C5—C8—H8B	109.5
P2—N2—P1	95.90 (17)	C5—C8—H8C	109.5
C5—N2—P1	132.7 (3)	H8A—C8—H8B	109.5
C5—N2—P2	131.3 (3)	H8A—C8—H8C	109.5
P1—N1—P2	95.51 (18)	H8B—C8—H8C	109.5
C1—N1—P1	132.2 (3)	C1—C3—H3A	109.5
C1—N1—P2	132.2 (3)	C1—C3—H3B	109.5
P1—N3—H3	110 (3)	C1—C3—H3C	109.5
C9—N3—P1	135.0 (3)	H3A—C3—H3B	109.5
C9—N3—H3	115 (3)	H3A—C3—H3C	109.5
P2—N4—H4	105 (4)	H3B—C3—H3C	109.5
C13—N4—P2	135.5 (3)	C9—C12—H12A	109.5
C13—N4—H4	119 (4)	C9—C12—H12B	109.5
C17—N5—C19	123.0 (5)	C9—C12—H12C	109.5
C17—N5—C18	120.8 (5)	H12A—C12—H12B	109.5
C18—N5—C19	116.2 (5)	H12A—C12—H12C	109.5
N2—C5—C6	109.4 (4)	H12B—C12—H12C	109.5
N2—C5—C7	108.6 (4)	C13—C16—H16A	109.5
N2—C5—C8	107.9 (4)	C13—C16—H16B	109.5
C7—C5—C6	109.8 (4)	C13—C16—H16C	109.5
C7—C5—C8	110.2 (5)	H16A—C16—H16B	109.5
C8—C5—C6	111.0 (4)	H16A—C16—H16C	109.5
C5—C6—H6A	109.5	H16B—C16—H16C	109.5
C5—C6—H6B	109.5	N5—C19—H19A	109.5
C5—C6—H6C	109.5	N5—C19—H19B	109.5
H6A—C6—H6B	109.5	N5—C19—H19C	109.5
H6A—C6—H6C	109.5	H19A—C19—H19B	109.5
H6B—C6—H6C	109.5	H19A—C19—H19C	109.5
H11A—C11—H11B	109.5	H19B—C19—H19C	109.5
H11A—C11—H11C	109.5	C13—C14—H14A	109.5
H11B—C11—H11C	109.5	C13—C14—H14B	109.5
C9—C11—H11A	109.5	C13—C14—H14C	109.5
C9—C11—H11B	109.5	H14A—C14—H14B	109.5
C9—C11—H11C	109.5	H14A—C14—H14C	109.5
N3—C9—C11	111.4 (4)	H14B—C14—H14C	109.5
N3—C9—C10	110.4 (4)	O1—C17—N5	125.7 (5)
N3—C9—C12	105.5 (4)	O1—C17—H17	117.2
C11—C9—C10	110.5 (5)	N5—C17—H17	117.2
C11—C9—C12	109.8 (4)	C1—C2—H2A	109.5
C10—C9—C12	109.2 (4)	C1—C2—H2B	109.5
N1—C1—C4	109.5 (4)	C1—C2—H2C	109.5
N1—C1—C3	109.0 (4)	H2A—C2—H2B	109.5
N1—C1—C2	108.1 (4)	H2A—C2—H2C	109.5
C4—C1—C3	109.9 (4)	H2B—C2—H2C	109.5
C4—C1—C2	110.8 (4)	N5—C18—H18A	109.5
C3—C1—C2	109.5 (4)	N5—C18—H18B	109.5
C9—C10—H10A	109.5	N5—C18—H18C	109.5
C9—C10—H10B	109.5	H18A—C18—H18B	109.5
C9—C10—H10C	109.5	H18A—C18—H18C	109.5
H10A—C10—H10B	109.5	H18B—C18—H18C	109.5
			
Se1—P1—N2—P2	107.86 (15)	P2—N1—C1—C4	40.6 (6)
Se1—P1—N2—C5	−75.0 (4)	P2—N1—C1—C3	160.8 (4)
Se1—P1—N1—P2	−108.19 (15)	P2—N1—C1—C2	−80.2 (5)
Se1—P1—N1—C1	69.0 (4)	P2—N4—C13—C15	−66.1 (6)
Se1—P1—N3—C9	9.6 (5)	P2—N4—C13—C16	175.0 (4)
Se2—P2—N2—P1	−109.36 (14)	P2—N4—C13—C14	58.0 (6)
Se2—P2—N2—C5	73.5 (4)	N2—P1—N1—P2	6.44 (18)
Se2—P2—N1—P1	106.70 (14)	N2—P1—N1—C1	−176.4 (4)
Se2—P2—N1—C1	−70.4 (4)	N2—P1—N3—C9	−124.4 (4)
Se2—P2—N4—C13	7.9 (5)	N2—P2—N1—P1	−6.47 (19)
P1—P2—N2—C5	−177.2 (5)	N2—P2—N1—C1	176.4 (4)
P1—P2—N1—C1	−177.1 (5)	N2—P2—N4—C13	140.0 (4)
P1—P2—N4—C13	−173.4 (4)	N1—P1—N2—P2	−6.51 (19)
P1—N2—C5—C6	36.4 (6)	N1—P1—N2—C5	170.6 (4)
P1—N2—C5—C7	156.1 (4)	N1—P1—N3—C9	142.7 (4)
P1—N2—C5—C8	−84.5 (5)	N1—P2—N2—P1	6.45 (19)
P1—N1—C1—C4	−135.6 (4)	N1—P2—N2—C5	−170.7 (4)
P1—N1—C1—C3	−15.3 (6)	N1—P2—N4—C13	−127.4 (4)
P1—N1—C1—C2	103.6 (5)	N3—P1—N2—P2	−118.1 (2)
P1—N3—C9—C11	57.2 (6)	N3—P1—N2—C5	59.0 (5)
P1—N3—C9—C10	−65.9 (6)	N3—P1—N1—P2	118.7 (2)
P1—N3—C9—C12	176.3 (4)	N3—P1—N1—C1	−64.2 (5)
P2—P1—N2—C5	177.1 (5)	N4—P2—N2—P1	117.9 (2)
P2—P1—N1—C1	177.1 (5)	N4—P2—N2—C5	−59.3 (4)
P2—P1—N3—C9	−170.7 (4)	N4—P2—N1—P1	−118.6 (2)
P2—N2—C5—C6	−147.5 (4)	N4—P2—N1—C1	64.2 (5)
P2—N2—C5—C7	−27.7 (6)	C19—N5—C17—O1	−179.1 (6)
P2—N2—C5—C8	91.7 (5)	C18—N5—C17—O1	0.9 (10)

### [1,3-Di-tert-butyl-2,4-bis(tert-butylamino)-1,3,2λ5,4λ5-diazadiphosphetidine-2,4-diselone-κ2Se,Se']diiodidomercury(II) N,N-dimethylformamide monosolvate Hydrogen-bond geometry (Å, º)

**Table:**

*D*—H···*A*	*D*—H	H···*A*	*D*···*A*	*D*—H···*A*
N3—H3···O1	0.82 (6)	2.18 (6)	2.986 (6)	165 (4)
N4—H4···O1	0.79 (5)	2.27 (5)	3.051 (5)	169 (6)
